# Development of Hybrid Surfaces with Tunable Wettability by Selective Surface Modifications

**DOI:** 10.3390/ma9030136

**Published:** 2016-02-26

**Authors:** Hyun-Joong Lee, Keun Park

**Affiliations:** 1 Graduate School of Mechanical Design and Robot Engineering, Seoul National University of Science and Technology, Seoul 01811, Korea; lhj8905@seoultech.ac.kr; 2 Department of Mechanical System Design Engineering, Seoul National University of Science and Technology, Seoul 01811, Korea

**Keywords:** wettability, superhydrophobic, hydrophilic, ultrasonic imprinting, electron beam, water collection, micropattern

## Abstract

Recent advances in micro/nano technology have driven artificial modifications of surface wettability by mimicking biological surfaces, such as superhydrophobic and water-harvesting surfaces. In this study, surface wettability of polycarbonate (PC) films was modified using various surface treatments: micropatterning using ultrasonic imprint lithography, fluorinate silane coating, and electron beam irradiation. To modify surface wettability selectively in a specified region, these three treatments were performed using profiled masks with the corresponding shapes. Various combinations of these treatments were investigated in terms of wettability changes, by measuring contact angle (CA). The semi-hydrophobic PC film (CA: 89.2°) was modified to create a super- hydrophobic state (CA: 155.9°) by virtue of the selective micropatterning and coating. The electron beam irradiation had an opposite effect, reducing the CA (48.2°), so that the irradiated region was modified to create a hydrophilic state. Two combinations of the proposed surface modifications made it possible to have a great difference in CA on a single surface (107.7°), and to have four different wetting states on a single surface. Various water-drop experiments proved that the developed hybrid surfaces were selectively wettable and showed water-collecting capability.

## 1. Introduction

These days, biological surfaces in nature have been received increasing interests in various academic and engineering fields [1,2,3]. Many studies have been performed to understand and imitate special functions of biological surfaces, and this knowledge has been applied to control surface wettability artificially by virtue of the recent advances in nanotechnology. One of the most popular applications is to develop superhydrophobic surfaces by mimicking the water-repellent characteristics of lotus leaves [4,5,6]. Another application is development of water harvesting surfaces by mimicking the fog-basking capability of beetles in the Namib Desert [7,8].

To develop superhydrophobic surfaces, various studies were carried out to reduce surface energy either by surface roughening or hydrophobic coating, or by combinations of these two approaches [9,10]. The surface roughening was conducted using various physical or chemical treatments, generally by fabricating micro/nano hierarchical structures via photolithography [11], capillary lithography [12], nanoparticle assembly [13], plasma treatment [14], chemical deposition [15], sol-gel processing [16], or micro/nanoscale patterning [17]. Hydrophobic surface coatings were conducted using low surface-energy materials: fatty acids [18]; organic silanes [19]; porous polymers [20]; and silicon compounds [21].

Among these treatments, micro/nanoscale patterning technologies have widely used to replicate rough structures because a number of soft substrates can be replicated efficiently out of hard molds. In general, the micro/nanoscale patterning can be categorized into two processes: thermal imprinting (hot embossing) and ultraviolet (UV) imprinting. The thermal imprinting uses preheated molds to replicate micro/nanoscale patterns on thermoplastic polymer substrates [22]. While this process has advantages of simple and economical setup, it has disadvantages of long cycle times to heat a mold before imprinting and to cool the mold after imprinting [23]. On the other hand, the UV imprinting uses UV-curable resins to transfer micro/nanoscale patterns at room temperature and low pressure [24]. This process has a limitation in its material usage (UV curable resin) although it has advantages in its capability of mass production and nanoscale patterning. Ultrasonic imprinting was recently developed to replicate micro/nano patterns on thermoplastic polymer using ultrasonic vibration energy, instead of the conventional thermal energy [25]. This process is advantageous in its short processing time and localized heating capability, which enabled further extension to repetitive patterning [26] or selective patterning [27] for developments of versatile functional surfaces.

To develop water-collecting surfaces, various studies were conducted to control surface wettability by developing an array of hydrophilic spots surrounded by hydrophobic or superhydrophobic backgrounds. To develop hydrophobic/superhydrophobic backgrounds, the aforementioned methods to reduce surface energy were applied according to the target material and the relevant surface characteristics. To develop an array of hydrophilic spots in the hydrophobic/superhydrophobic background, pattern-wise surface hydrophilization was performed using various treatments including plasma deposition [28,29,30], UV radiation through photomasks [31,32,33], polydopamine functionalization [34,35], and surface oxidization [36].

The aim of this study was to develop hybrid surfaces with tunable wettability, on which the surface wettability can be differentiated over a large area, rather than over an array of microscale spots. To develop these hybrid surfaces efficiently, three facile treatments for selected regions were used: ultrasonic imprinting, fluorinate coating, and electron beam (e-beam) irradiation. The selective ultrasonic imprinting was applied in combination with selective fluorinate coating, in order to modify a semi-hydrophobic polycarbonate (PC) surface to a super- hydrophobic state by reducing its surface energy. To increase the surface energy for hydrophilic modification, an electron beam was used to selectively irradiate the PC film that was covered with a profiled mask. Various types of functional surfaces with different wettability were then developed and analyzed by changing the mask shapes for the selective imprinting, coating, and irradiation.

## 2. Materials and Methods

### 2.1. Materials

As a target material, PC films (GP-1000L, LG Chemical Co., Seoul, Korea) 0.3 mm thick were prepared. The glass transition temperature of the PC film was 150 °C. As a micromold for ultrasonic imprinting, a nickel stamp was fabricated as described in Section 2.2.1, and was assembled with an AISI-1045 steel mold base. Aluminum alloy (AA-1050, Novelis Korea Co. Ltd., Seoul, Korea) sheets were used as mask films (0.4 mm thick). These mask films were fabricated by waterjet cutting, in order to have an outline similar to the musical sharp (#) sign for the selective ultrasonic imprinting and the selective fluorinate coating: a negative mask for the selective imprinting and a positive mask for the selective coating. As the ultrasonic horn material, high-strength aluminum alloy (AA7075-T651, Dongyang Aluminum Co. Ltd., Siheung, Korea) was used due to its high mechanical strength and ultrasonic transmission capability. As a fluorinate coating material, trichloro (1H, 1H, 2H, 2H-perfluorooctyl) silane (Sigma-Aldrich Corp., St. Louis, MO, USA) was used. To use the electron beam to irradiate only a selected region, SS440 steel sheets 4.0 mm thick were fabricated to have a negative sharp shape, and were used as a mask film for e-beam irradiation.

### 2.2. Selective Micropatterning

#### 2.2.1. Preparation of a Micropatterned Mold

**Figure 1:**
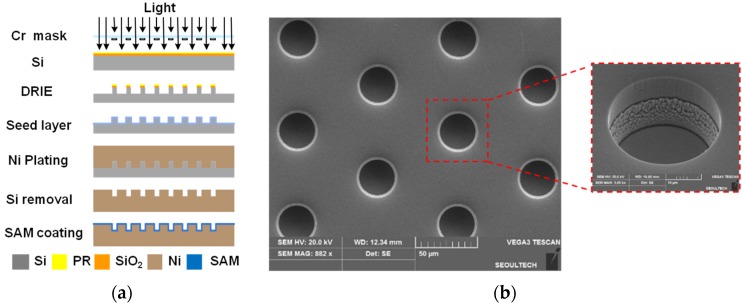
Fabrication of the nickel stamp: (**a**) fabrication procedure; and (**b**) SEM photograph of the fabricated nickel stamp.

To develop hydrophobic micropatterns, a number of circular pillars (30 μm diameter and 20 μm height) were designed to be placed at a 75 μm pitch with a hexagonal arrangement, which ensures high CA in the Cassie’s regime [37]. A micromold was then fabricated to have a number of microscale holes, as a negative shape of the designed pillar pattern. Figure 1a illustrates the fabrication process of the micromold. At first, the photolithography process was conducted on a silicon master using a chrome mask. The deep reactive ion etching (DRIE) process was followed to develop positive micropillar arrays on the surface of the silicon master. The sputtering process was performed to deposit a copper seed layer on the surface of the fabricated silicon master. A nickel stamp was then fabricated using the electroforming process, from which a number of microhole arrays were developed as a negative shape. After removing the silicon master, a self-assembled monolayer (SAM) was deposited on the surface of the nickel stamp in order to facilitate demolding after imprinting. Figure 1b shows a scanning electron microscope (SEM) photograph of the developed microscale holes on the stamp surface. This nickel stamp was then assembled with a steel mold base, from which a micromold for ultrasonic imprinting was prepared.

#### 2.2.2. Selective Micropattern Replication

**Figure 2:**
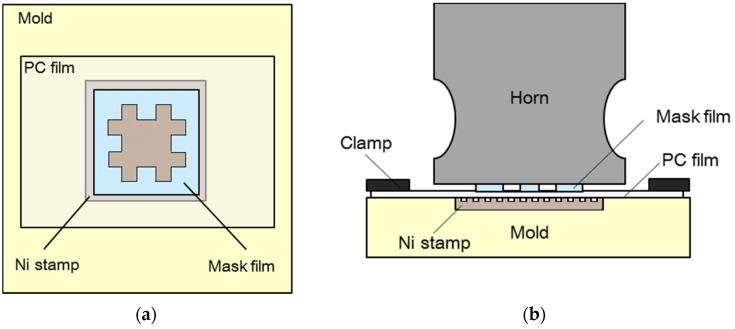
Configuration of selective ultrasonic imprinting using a profiled mask film: (**a**) top view; and (**b**) side view.

To replicate micropatterns on a selected area of a PC film, ultrasonic imprinting was performed using a mask film. Figure 2a shows a top view of the imprinting section, including a negative ‘#’-shaped mask film, with a size of 20 × 20 mm^2^. This mask film was placed between the horn surface and target polymer film, as illustrated in Figure 2b. The prepared target PC film was 80 × 40 mm^2^, and the micropatterned stamp was 22 × 22 mm^2^. The horn was designed to have a rectangular cross-section, with an outlet size of 42 × 39 mm^2^, in order to cover the entire micropatterned region, as shown in Figure 2a. Selective micropattern replication was then performed using an ultrasonic imprinting system with a frequency of 19.8 kHz and power of 1.4 kW. Ultrasonic vibration time, holding time, holding pressure, and mold temperature were set to 4.5 s, 3.0 s, 0.6 MPa, and 90 °C, respectively. Under this experimental setup, ultrasonic waves were transferred from the ultrasonic horn to the target film through the profiled mask film, so that the micropatterns were replicated only in the masked region [27].

### 2.3. Selective Fluorinate Coating

**Figure 3:**
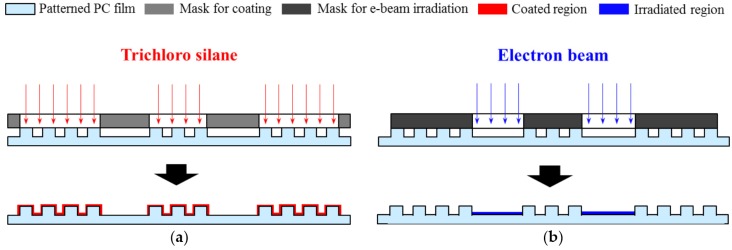
Surface treatments for the selected regions: (**a**) fluorinate coating for the negative region; and (**b**) e-beam irradiation for the positive region.

For selective fluorinate coating, trichloro (1H, 1H, 2H, 2H-perfluorooctyl) silane was coated onto the PC films. The selectively-patterned PC films were placed in a vacuum chamber, covered with positively shaped masks, as shown in Figure 3a. The silane solution was also placed in the vacuum chamber, and was evaporated under 5 × 10^−4^ Torr pressure for five minutes. Since this fluorinate coating was performed selectively on the micropatterned region only, it was expected that the surface energy of the corresponding region would be further reduced and could, thus, be differentiated from the remaining region.

### 2.4. Electron Beam Irradiation

The electron beam was used to irradiate a number of selectively patterned PC films. In the irradiation process, the same number of negative SS440 masks was covered on the patterned films so that the electron beam should irradiate only the corresponding positive regions, as shown in Figure 3b. The irradiation experiments were performed using a 0.2 MeV electron accelerator at the Korea Atomic Energy Research Institute (KAERI, Daejeon, Korea). The irradiation current and time were set to 0.4 mA and 150 s, respectively. Since this e-beam irradiation was performed selectively in the positive region only, it was expected that the surface energy of the corresponding region would be increased, so that the region would be hydrophilized.

### 2.5. Chracterization

During the ultrasonic imprinting process, the temperature distributions of imprinted PC films were measured using an infrared thermal imaging system (FLIR E50, FLIR Systems Inc., Boston, MA, USA). A scanning electron microscope (SEM, VEGA3, Tescan, Brno, Czech) was used to observe the fabricated micromold and the replicated micropatterns. Surface characteristics in the selectively coated regions were analyzed using an X-ray photoelectron spectroscope (XPS) and energy dispersive spectroscopy (EDS). The chemical compositions of the coated and irradiated regions were characterized using XPS (Multilab. ESCA 2000, VG Microtech. Co., East Grinstead, West Sussex, UK) analysis. The chemical composition of the coated micropillars was characterized using EDS (VEGA3, Tescan, Brno, Czech, attached to the SEM) analysis. CAs were measured using a goniometer (CAM-200, KSV Instrument Ltd., Helsinki, Finland) with deionized water drops of 3 μL, five times for each condition.

## 3. Results

### 3.1. Surface Hydrophobitization Using Selective Micropatterning

**Figure 4:**
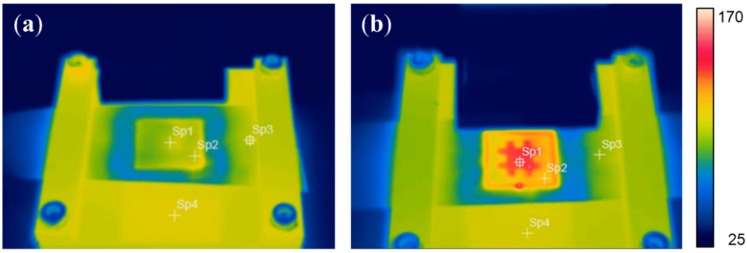
Temperature distributions after ultrasonic imprinting: (**a**) normal imprinting; and (**b**) selective imprinting.

Selective ultrasonic imprinting was conducted for PC films, using the fabricated mask and micropatterned mold. To investigate the localized heating effect of the selective ultrasonic imprinting, the temperature distribution in the imprinting region was measured after the selective imprinting, and was compared with that of the normal ultrasonic imprinting. Figure 4a,b show the measured temperature distributions for normal and selective imprinting, respectively. The maximum temperature of the selectively-imprinted sample was as high as 153.8 °C, while that of the normally-imprinted sample was just 102.2 °C. This indicates that the selective transmission of ultrasonic wave results in localized heating in the masked region, raising its surface temperature higher than the glass transition temperature (150 °C). Therefore, the PC film could be softened locally and rapidly, only in 4.5 s vibration time.

**Figure 5:**
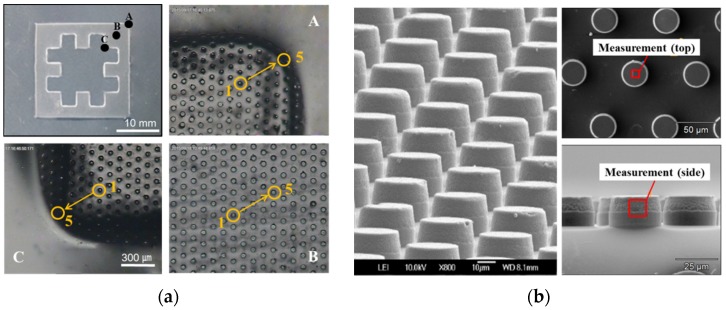
Replicated micropatterns by selective ultrasonic imprinting: (**a**) replicated micropatterns in different regions; and (**b**) SEM images of the replicated micropillars in Region B.

Figure 5a shows microscope images of various regions (Regions A to C) of the selectively-patterned PC film. Figure 5b shows SEM images of the replicated micropatterns in Region B, showing that a number of micropillars were developed successfully. While these micropatterns were well replicated in the masked area (Region B), they were not replicated in the unmasked area, as shown in the images of the boundary regions (Regions A and C). It can be seen that the corners of the boundary regions were not sharp but curved because the corner radii of the fabricated mask were 482.5 ± 37.7 μm due to the resolution of the waterjet cutting.

To investigate the pattern transfer quality, pattern heights were measured for five replicated pillars in the three regions, as marked in Figure 5a. In the boundary regions (Regions A and C), the measured pillars were numbered from inside (Pillar 1) to outside (Pillar 5). The measured pattern height was 19.76 ± 0.31 μm in the masked region (Region B), showing a high and uniform replication quality. In contrast, the boundary regions showed lower replication heights and larger deviations: 14.88 ± 5.24 μm in Region A and 11.14 ± 6.17 μm in Region C. The reason of such low transfer quality is that some micropatterns were replicated even in the unmasked region due to the heat transfer effect [38]. In Region A, the height of Pillar 1 was 19.7 μm while that of Pillar 5 was only 7.1 μm. In Region C, the height of Pillar 1 was 19.1 μm while that of Pillar 5 was only 5.2 μm. This indicates that the pattern transfer quality shows a rapid change from the innermost region (higher than 95%) to the boundary region (lower than 35%). Therefore, it can be noted that this rapid and localized degeneration in the boundary regions does not affect the selective wetting characteristics of the developed hybrid surface because incompletely-developed micropatterns could not increase surface hydrophobicity significantly [17].

### 3.2. Surface Hydrophobitization Using Selective Fluoriate Coating

**Figure 6:**
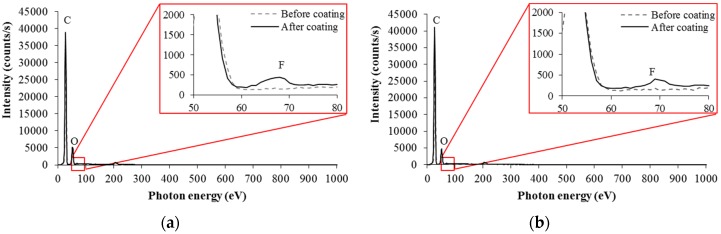
EDS analysis results for the coated micropillars: (**a**) top region; and (**b**) side region.

Selective fluorinate coatings were added to further reduce the surface energy of the selectively-patterned PC films. To investigate the change in chemical composition of the developed micropatterns, EDS analyses were performed for the top and side regions of a coated micropillar, as marked in Figure 5b. Figure 6a,b represent the resulting EDS spectra for the top and side regions, showing peaks at 0.69 keV in both cases. These peaks prove the existence of fluorine in those regions, which means that hydrophobic silane was stably coated onto the surfaces of the developed micropillars.

**Figure 7:**
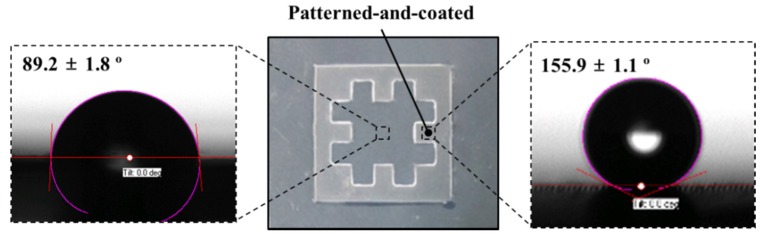
Comparison of CAs for the pure region and patterned-and-coated region.

To investigate the change of wettability in the patterned-and-coated region, CA was measured and compared with that in the pure region. Figure 7 shows photographs of the water droplet shapes in the pure and patterned-and-coated regions. It can be seen that the pure region shows a semi-hydrophobic state (CA: 89.2° ± 1.8°), while the patterned-and-coated region shows a super-hydrophobic state (CA: 155.9° ± 1.1°). This indicates that the micropatterning with fluorinate coating changed a semi-hydrophobic PC surface to a superhydrophobic state, showing an increase in the CA of more than 66°.

### 3.3. Surface Hydrophlization Using Electron Beam Irradiation

**Figure 8:**
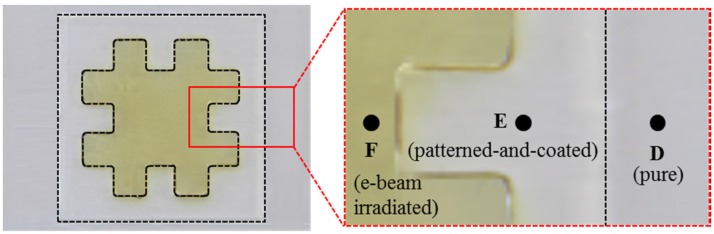
Photographs and detailed view for the selectively irradiated sample. Regions D, E, and F denote pure, patterned-and-coated, and e-beam irradiated regions, respectively.

**Figure 9:**
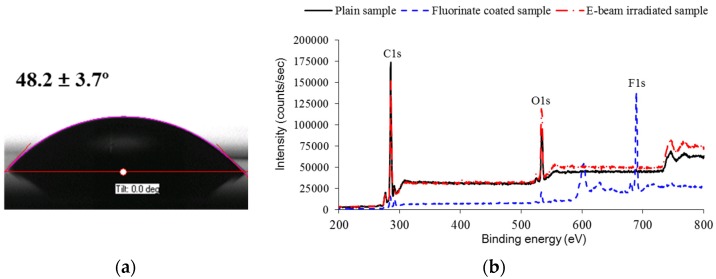
Analyses of the e-beam irradiated region (F): (**a**) CA measurement; and (**b**) XPS analysis.

The e-beam was used to irradiate the selectively-patterned PC films, using the fabricated mask with a negative shape. Figure 8 shows the selectively irradiated PC sample, where the color of the irradiated region has changed to yellowish. This color change is supposed to originate from electrons and free radicals captured during e-beam irradiation [39]. Figure 9a shows the water droplet shapes in the e-beam irradiated region, showing that its CA was reduced to 48.2° ± 3.7°. To compare the chemical composition changes according to each treatment, XPS analyses were performed in the pure (D), patterned-and-coated (E), and e-beam irradiated regions (F). Figure 9b shows XPS spectra in these three regions, and their quantitative results are compared in Table 1. It can be seen that the pure region showed C1s and O1s peaks only while the coated region showed an additional F1s peak. The existence of fluorine acted to increase surface hydrophobicity, in addition to the hydrophobic micropatterns. In the case of the irradiated region, the carbon content decreased by 8.1%, while the oxygen content increased by 5.9%. This result indicates that the hydrophobic group (C-C) decreased while the hydrophilic group (C-O) increased. This increase of hydrophilic groups then successfully modified the semi-hydrophobic PC surface into hydrophilic state, by reducing the corresponding CA as much as 41°.

## 4. Discussion

### 4.1. Investigation of Selective Wettability

**Figure 10:**
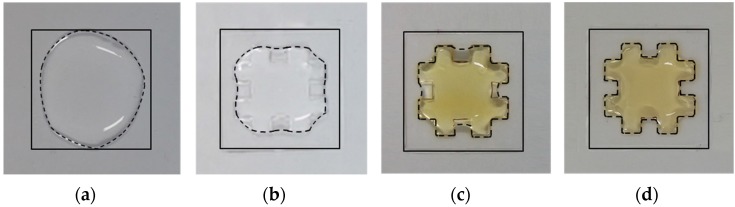
Comparison of water dispersion shapes at different treatment stages: (**a**) pure PC sample; (**b**) after selective micropatterning; (**c**) after e-beam irradiation; and (**d**) after fluorinate coating.

Based on the results in the previous section, it can be seen that the surface energy of a single surface could be differentiated significantly; the difference in CA between the superhydrophobic and hydrophilic regions was as high as 107.7°. To investigate selective wettability of the developed hybrid surface, the water dispersion characteristics were evaluated by dropping large amount of water drops (200 μL). Figure 10 compares the resulting water dispersions at each intermediate stage: pure PC sample (Figure 10a); after selective micropatterning (Figure 10b); after e-beam irradiation (Figure 10c); and after fluorinate coating (Figure 10d). In each case, the boundary for dispersed water was marked with a dashed line. It can be seen that the boundary shapes changed close to the desired shape as each treatment (selective micropatterning, e-beam irradiation, and fluorinate coating) was performed; the water remained only in the irradiated hydrophilic region after all treatments, as shown in Figure 10d. Therefore, a hydrophilic region surrounded by a superhydrophobic region could be wetted selectively, even over such a large area.

### 4.2. Water Collection of the Developed Hybrid Surface

**Figure 11:**
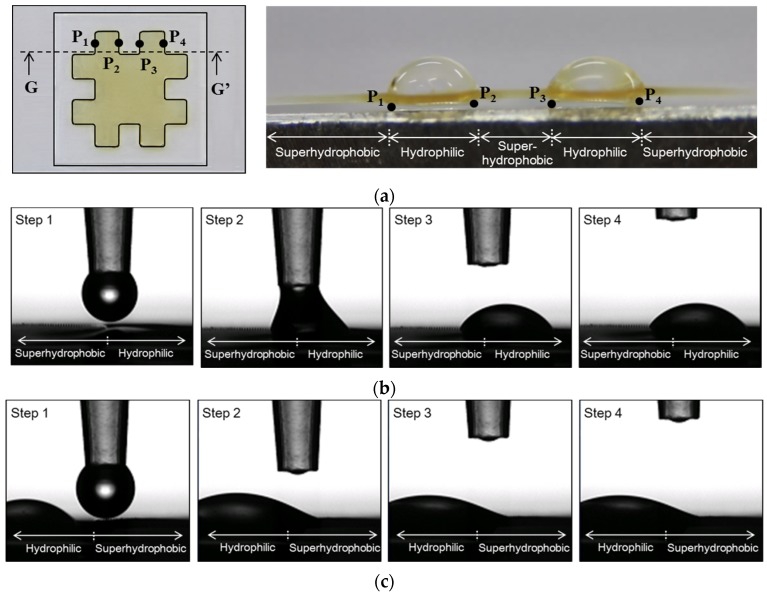
Movements of water droplets at the superhydrophobic-hydrophilic interface points. (**a**) Definition of drop points and the resulting droplet shapes (at section GG’); (**b**) Water droplet movement at P_1_; (**c**) Water droplet movement at P_2_.

To gather additional information, a water collection test was conducted for the developed superhydrophobic–hydrophilic hybrid surface. To investigate the water collection characteristics, water droplets were dropped at the superhydrophobic–hydrophilic interface points (P_1_ to P_4_ in Figure 11a), with a droplet volume of 3 μL. At first, a water droplet was placed at P_1_, an interface of superhydrophobic-hydrophilic regions. Figure 11b show the movement of the water droplet, from the superhydrophobic region to the hydrophilic region. The second droplet was then placed at P_2_, an interface of hydrophilic–superhydrophobic regions. This droplet moved to the left as shown in Figure 11c, and merged into the existing droplet in the hydrophilic region. The same procedure was repeated for the points P_3_ and P_4_. Consequently, four water droplets at four boundary points could be collected in two hydrophilic regions, as shown on the right side of Figure 11a. This result indicates that the developed hybrid surface has water-collecting capability caused by differentiating the surface energy significantly on a single surface.

### 4.3. Development of Hybrid Surface with Tunable Wettability

**Figure 12:**
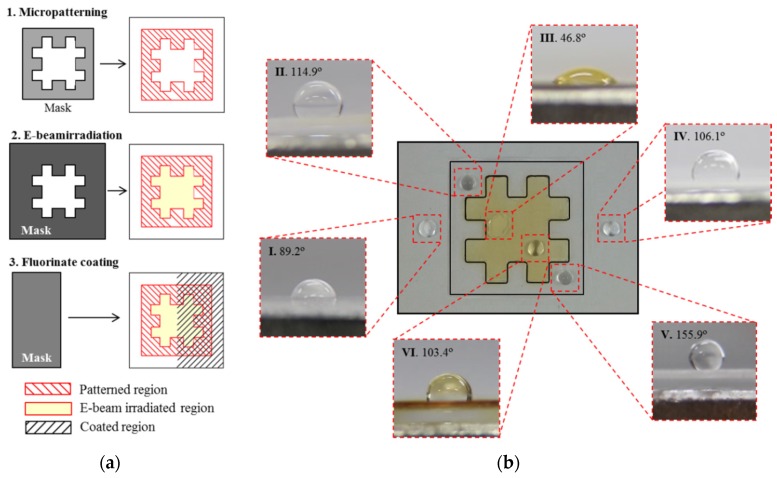
Development of a hybrid surface with a combination of three surface treatments: (**a**) stepwise preparation of the three treatments; and (**b**) CAs measured in the six different regions.

In this section, a different type of hybrid surface was developed by combining the aforementioned three treatments with different masks. Figure 12a illustrates the stepwise preparation procedure of the three treatments, with an order of micropatterning, e-beam irradiation, and fluorinate coating. The selective micropatterning and e-beam irradiation were performed using the same masks as those in the previous sections. In contrast, the selective fluorinate coating was conducted using a rectangular mask that covered the left half region, so that only the right half region was coated.

The developed hybrid surface can then be divided into six different regions, as shown in Figure 12b: pure region (Region I); micropatterned region (Region II); e-beam irradiated region (Region III); coated region (Region IV); patterned-and-coated region (Region V); and irradiated-and-coated region (Region VI). CAs were measured in the six regions, which showed different wetting states: hydrophilic in Region III (CA: 46.8°), semi-hydrophobic in Region I (CA: 89.2°), superhydrophobic in Region V (CA: 155.9°), and hydrophobic in Region II (CA: 114.9°), Region IV (CA: 106.1°), and Region VI (CA: 103.4°). Therefore, six different regions with four different wettability states were obtained by combining the proposed three treatments effectively.

## 5. Conclusions

In this study, surface wettability of PC films was modified by three different treatments: ultrasonic imprinting, fluorinate coating, and e-beam irradiation. Micropatterning using ultrasonic imprinting and fluorinate silane coating were performed to reduce surface energy, from which the semi-hydrophobic PC film (CA: 89.2°) could be modified into a superhydrophobic state (CA: 155.9°). The e-beam irradiation was performed to increase surface energy, from which the PC film could be modified into a hydrophilic state (CA: 48.2°). These three treatments were conducted selectively in specified regions using the profiled masks with the corresponding shapes. As a result, a hydrophilic–superhydrophobic hybrid surface could be developed, in which the difference in CAs between adjacent superhydrophobic and hydrophilic regions could be as great as 107.7°. This selective surface modification was further extended by making different combinations of surface treatments with different masks, from which six different regions with four different wettability states could be obtained on a single surface.

Through various water-drop experiments, it was verified that this hybrid surface, which exhibits large differences in CA, was selectively wettable and had water-collecting ability. Considering that the three treatments are selectively applicable over a large area with low processing time and costs, it can be concluded that the proposed approach is useful for developing versatile functional surfaces with different wettability. Therefore, the proposed approach can be utilized to develop various functional surfaces with tunable wettability, such as self-cleaning, anti-fogging, anti-icing surfaces. Furthermore, these functional surfaces can be extended to develop water collecting devices, biological devices, and partially-wettable products in which we can select wetted or non-wetted regions according to their use.

## Figures and Tables

**Table 1:** Comparison of XPS surface analysis results in various regions with different treatments

Elemental Components	Pure (D)	Coated (E)	Irradiated (F)
C1s	84.1	42.9	76.0
O1s	15.9	8.3	21.8
F1s	–	44.7	–
N1s	–	–	2.2
Si2p	–	4.1	–
